# New Observations on a Low‐Grade Diffusely Infiltrative Tumour, *SMARCB1* Mutant, Arguing for a New Tumour Type

**DOI:** 10.1111/nan.70098

**Published:** 2026-09-07

**Authors:** Alice Métais, Giorgia Antonia Simboli, Marc Barritault, Fabrice Chrétien, Julien Masliah‐Planchon, Alessandro Moiraghi, Johan Pallud, Laure Thomas, Pierre Leblond, Alexandre Bani‐Sadr, Raphaël Saffroy, Arnault Tauziède‐Espariat, Alexandre Vasiljevic, Pascale Varlet

**Affiliations:** ^1^ Department of Neuropathology GHU Paris ‐ Psychiatry and Neuroscience, Sainte‐Anne Hospital Paris France; ^2^ Translational Research in Neuroscience Lab Institut Imagine, UMR‐1163, Université Paris Cité Paris France; ^3^ Clinical Pathology Service, Department of Diagnostics Geneva University Hospitals HUG Geneva Switzerland; ^4^ Department of Pathology, Molecular Biology of Tumors Hospices Civils de Lyon Groupement Hospitalier Est – HCL Bron France; ^5^ Université Paris Cité Paris France; ^6^ Laboratory of Somatic Genetics Institute Curie Hospital Paris France; ^7^ Service de Neurochirurgie GHU‐Paris Psychiatrie et Neurosciences, Site Sainte Anne Paris France; ^8^ Department of Neuro‐Oncology East Group Hospital, Hospices Civils de Lyon Bron France; ^9^ Institute of Pediatric Hematology and Oncology (IHOPe) Centre Léon Bérard Lyon France; ^10^ Childhood Cancer & Cell Death Team (C3 Team), Consortium South‐ROCK Centre de Recherche en Cancérologie de Lyon (CRCL), INSERM 1052, CNRS 5286 Lyon France; ^11^ Department of Neuroradiology East Group Hospital, Hospices Civils de Lyon Bron France; ^12^ Department of Biochemistry and Oncogenetic Paul Brousse Hospital Villejuif France; ^13^ Institute of Psychiatry and Neuroscience of Paris (IPNP) Université Paris Cité, INSERM U1266 Paris France; ^14^ Pathology Department, Reference Center for Rare Pituitary Diseases HYPO ‘Groupement Hospitalier Est’ Hospices Civils de Lyon Bron France

## Abstract

Two new cases of low‐grade diffusely infiltrative tumour (LGDIT), SMARCB1 mutant, are described in an 18‐year‐old and a 50‐year‐old male, both with supratentorial lesions, characteristic rhabdoid histology on a myxoid‐collagenous background, and complete INI1 loss. Both tumours showed homozygous SMARCB1 deletion and clustered with previously reported LGDIT on t‐SNE analysis, in proximity to ATRT‐MYC. These observations reinforce the distinct clinicopathological profile of LGDIT and support its consideration as a provisional CNS tumour type.

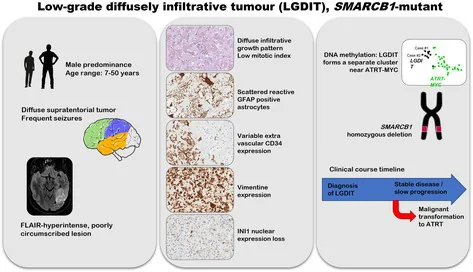

Recent studies have reported very rare, low‐grade, diffusely infiltrative tumours (LGDIT), *SMARCB1*‐mutant, expanding the spectrum of central nervous system (CNS) *SMARCB1*‐deficient tumours [1, 2, 3]. Despite their indolent clinical behaviour compared with aggressive atypical teratoid and rhabdoid tumours (ATRTs), malignant transformation of LGDIT into ATRT has been described and both share a similar DNA‐methylation profile, particularly close to the ATRT‐MYC subgroup [1, 4]. Here, two cases are reported whose morphological, immunophenotypic, molecular and epigenetic profiles were consistent with LGDIT, and a potential qualification as a provisional tumour type for the World Health Organization (WHO) classification of CNS tumours is discussed, according to cIMPACT‐NOW update 10 criteria [5].

The first patient was an 18‐year‐old male admitted for attentional and behavioural deficits. Brain MRI revealed a hyperintense bifrontal lesion on FLAIR imaging, with heterogeneous gadolinium enhancement, predominantly in the right frontal lobe (Figure 1). Hyperdensities on the computed tomography suggested calcification. The biopsy showed a moderately cellular, diffusely infiltrative tumour on a myxoid and collagenous background. The tumour was composed of spindle‐shaped cells and small round cells with a rhabdoid or oligo‐like appearance, with elongated or rounded nuclei and moderate atypia. Reactive astrocytes and degenerative neurons were scattered throughout the lesion. No honeycomb appearance, microvascular proliferation or necrosis was observed. Mitotic activity was low with 2 mitoses per 3 mm^2^. On immunohistochemistry (Figure S2), tumour cells were negative for GFAP, OLIG2 and myogenin. A subset of cells showed CD34 extravascular expression. MIB1 labelling index was around 5%. There was no expression of IDH1 R132H or H3K27M mutant proteins, no nuclear p53 accumulation and nuclear expression of ATRX and H3K27me3 was retained. Copy number variation (CNV) analysis from DNA‐methylation data showed a homozygous *SMARCB1* deletion, confirmed by whole‐exome sequencing (WES), which correlated with loss of nuclear SMARCB1/INI1 expression in the tumour cells (Figure 1). WES identified only a few variants of unknown significance (VUS): *MAPK1* with loss of heterozygosity (LOH) c.655G > A p.(Ala219Thr) with a variant allele frequency (VAF) of 41%; *KMT2D* with LOH c.7270C > T p.(Pro2424Ser) with a VAF of 40%; *CDKN2C* c.263C > T p.(Thr88Ile) with a VAF of 21%. No fusion transcripts were detected (Arriba version 2.0.0; Fusion Catcher version 1.20; StarFusion version 1.9.1). DNA methylation profiling, using the Heidelberg classifier v12.8, suggested the ATRT methylation class (calibrated score 0.86) and the subclass of ATRT‐MYC (calibrated score 0.58), and the t‐SNE analysis showed clustering with previously described cases of LGDIT (Figure 1). These findings supported the diagnosis of LGDIT. Adjuvant chemotherapy (carboplatin/etoposide/cyclophosphamide) was given for 2 months, followed by focal radiotherapy at 54 Gy (Figure S1). Clinical and radiological follow‐up at 26 months showed clinical stability and slow radiological progression compared with the 14‐month post‐operative MRI. The patient eventually died at 31 months of follow‐up due to progression.

**FIGURE 1:**
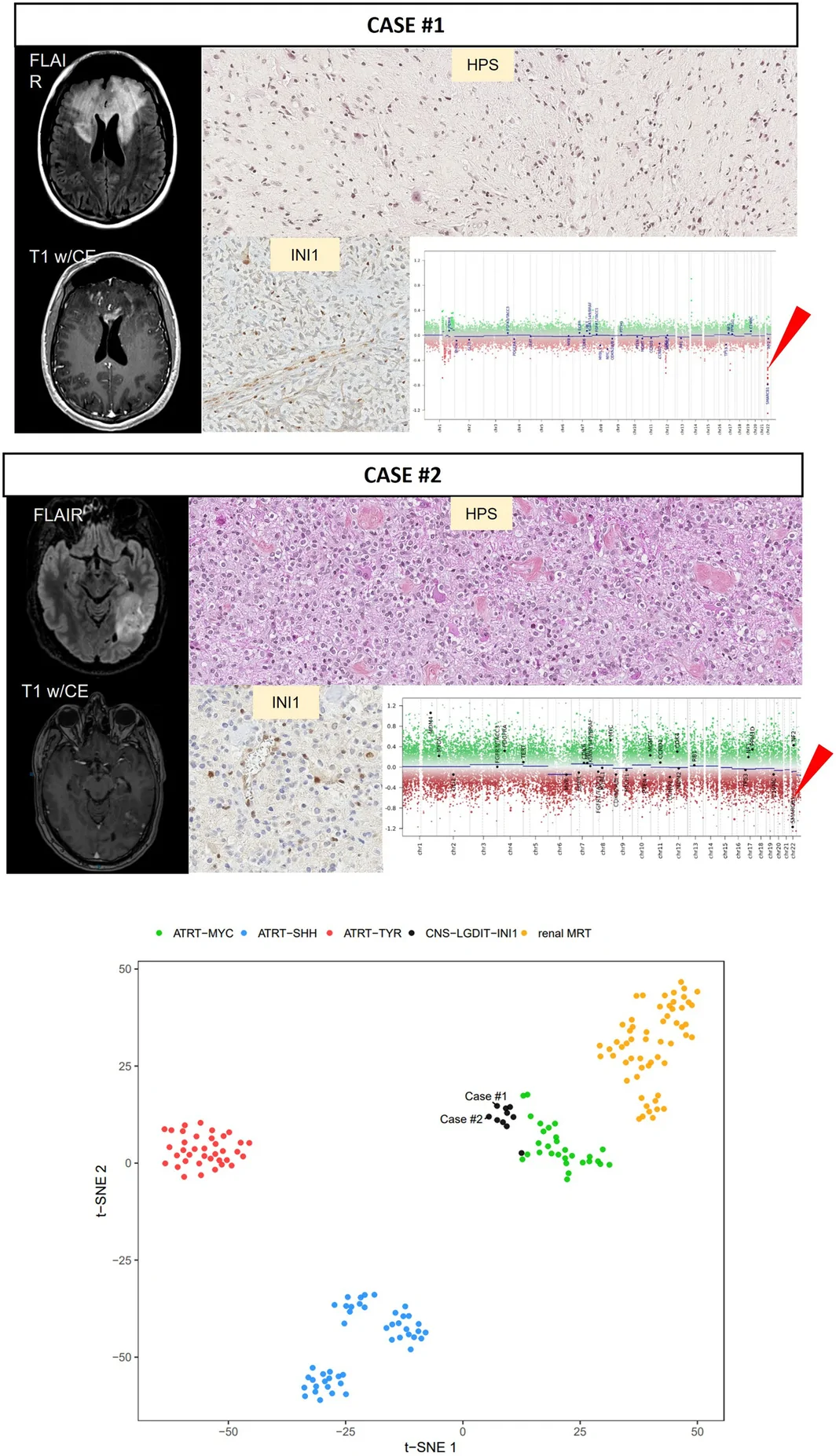
Radiological, histopathological, immunohistochemical, copy number variation plot of cases #1 and #2 and tSNE‐Analysis For each case, axial T1‐weighted post‐contrast and FLAIR magnetic MRI images are displayed alongside histopathological sections stained by haematoxylin eosin saffron (HPS) and anti‐INI1 immunohistochemical stains. Copy number variation profiles were obtained from Illumina EPIC V2 DNA methylation analysis. On t‐SNE analysis, DNA methylation profiles of the two case groups with previously published LGDIT, *SMARCB1*‐mutant (*N* = 8), in comparison to 29 ATRT MYC, 46 ATRT SHH, 37 ATRT TYR and 58 renal MRT.

The second patient was a 50‐year‐old male admitted for epileptic seizures. MRI showed an intra‐axial left parietal tumour with heterogeneous gadolinium enhancement on T1 and hyperintensity in T2 FLAIR (Figure 1). Neuropsychological assessment allowed awake surgery, resulting in sub‐total tumour resection. Histologically (Figure 1), there was a moderately cellular, diffusely infiltrative tumour involving cortex and juxtacortical white matter on a myxoid and collagenous background, with scattered reactive astrocytes and dystrophic neurons. Tumour cells were moderate to small in size, oligo‐like or rhabdoid, with moderate atypia. Mitotic activity was low, with up to 2 mitoses per 2 mm^2^, and no microvascular proliferation or necrosis was seen. Immunohistochemically, the tumour cells were negative for GFAP, OLIG2, synaptophysin, chromogranin A, MAP 2, NeuN, pan‐cytokeratin, CD45, smooth muscle actin, desmin, myogenin, HMB45, SOX10, CD163 and CD1a. Only a subset of tumour cells expressed vimentin and CD34 (Figure S2). Mutant IDH1 R132H, H3G34R and H3K27M proteins were not expressed, and nuclear ATRX and H3K27me3 expression was retained. There was a complete loss of INI1 nuclear expression in the tumour cells (Figure 1), consistent with homozygous *SMARCB1* deletion, confirmed by FISH, next‐generation sequencing (NGS) and CNV data from methylation profiling, despite suboptimal sample quality. NGS also detected a *KIT* VUS c.1588G > A p.(Val530Ile) (VAF 49%). No fusion transcript was identified. The Heidelberg classifier v12.8 did not provide a confident class assignment, likely due to sample quality, but on t‐SNE analysis, the case clustered with previously described LGDIT, supporting the diagnosis. No adjuvant oncological treatment was given. At 24‐month follow‐up, the patient remained clinically and radiologically stable, with unchanged residual tumour on MRI.

To date, 15 cases of LGDIT have been reported, including the two presented here, across six independent publications (Supplementary Table 1) [1, 2, 3, 6, 7, 8]. The aggregated data suggest a relatively consistent pattern of adolescent and young adult predominance (median 19 years, range 7–50 years), clear male predominance, and mainly supratentorial location, with epilepsy as a frequent presenting symptom. However, the clinical course is heterogeneous, ranging from prolonged stability to death within 3 years, and treatment strategies have varied considerably [2, 3]. DNA‐methylation profiling consistently places LGDIT in a separate cluster, close to ATRT‐MYC and desmoplastic myxoid tumour, *SMARCB1*‐mutant (DMT) [9], reflecting the dominant impact of SWI/SNF complex deficiency on the methylation landscape and of the cell of origin [1, 2]. This molecular distinction could be reinforced by transcriptomic studies, as has been observed for molecular subgroups of ATRT, but could also be related to lower tumour content of the samples and to contaminating nonneoplastic cells. At the molecular level, LGDIT appears to be characterised by a single genetic change, namely, a homozygous deletion of *SMARCB1* in 100% of the reported cases so far. This contrasts with other INI1‐deficient tumours, such as ATRT and DMT, which can show a broader spectrum of alterations (e.g., single‐nucleotide variants or small indels). Additional variants and small deletions of uncertain significance have been described but are not recurrent and are of uncertain significance [1, 2, 3]. No additional pathogenic variants were reported, though sequencing methods were limited. Importantly, LGDITs differ from secondary ATRT arising in meningioangiomatosis (MAM) or glioneuronal tumours, the latter being circumscribed lesions typically harbouring *BRAF* alterations [10, 11, 12]—two features absent in LGDIT. Across series, LGDIT is characterised by a moderately to highly cellular, diffusely infiltrative growth pattern, often poorly circumscribed, composed of monomorphic rhabdoid or oligo‐like cells in a myxoid‐collagenous matrix, with generally low mitotic activity (although up to 5 mitoses per 10 high‐power fields and a Ki‐67 index of 15% have been reported in the series by Zhao et al. [7]) and absence of frank microvascular proliferation or necrosis. Immunohistochemically, tumour cells lack glial lineage markers (GFAP, OLIG2), show focal CD34 and vimentin expression and consistently exhibit complete loss of INI1 nuclear staining. While no single feature is specific, the combination is distinctive and reproducible. The largest series of LGDIT so far included six patients, and the fact that cases have been reported worldwide reflects the rarity of the tumour while suggesting that underdiagnosis is likely. In light of cIMPACT‐NOW update 10, we systematically evaluated LGDIT against the proposed criteria for recognition of new CNS tumour types and have summarised the findings in Table 1 [5].

**TABLE 1:** Evaluation of low‐grade diffusely infiltrative tumour (LGDIT), *SMARCB1*‐mutant, against the cIMPACT‐NOW update 10 criteria for defining new central nervous system tumour types [5].

cIMPACT‐NOW criteria	Argument	Assessment
1—Clinical phenotype	The clinical phenotype is partly consistent, but outcome and treatment response remain markedly heterogeneous.	Partially met
2—Large‐scale molecular profiling	DNA methylation profiling places LGDIT in a separate cluster, although the available reference cohorts remain small.	Not yet fully met
3—Associated molecular profile	Homozygous *SMARCB1* deletion is the defining alteration, but no consistent secondary genetic event separates LGDIT from other *SMARCB1*‐deficient tumour types.	Not independently met
4—Microscopic and immunohistochemical features	The histological and immunophenotypic features are not specific but the combination is distinctive and reproducible.	Met
5—Independent publications	Although the published series remain small, LGDIT has now been reported across multiple independent studies.	Supports consideration as a provisional tumour type under the exceptional clause for very rare entities
6—cIMPACT‐NOW committee approval	Not yet applicable.	Not yet applicable

Two possible nosological interpretations can be proposed regarding the position of LGDIT. The first is that it represents a morphological variant of ATRT‐MYC. This view is supported by the consistent *SMARCB1* homozygous deletions, rhabdoid morphology and occasional progression to ATRT at recurrence, but is challenged by the distinct age distribution, infiltrative growth pattern and initial indolent course. An alternative interpretation is that LGDIT could be to ATRT‐MYC what cribriform neuroepithelial tumour (CRINET) is to ATRT‐TYR: an indolent, low‐grade counterpart within the same epigenetic lineage. Additional data with integrated histological, molecular and longitudinal data are required to resolve whether LGDIT should be formally recognised as a new CNS tumour type or considered an extremely rare low‐grade variant of ATRT‐MYC, with potential malignant evolution. The reported management of LGDIT has been heterogeneous, ranging from observation alone to adjuvant chemotherapy borrowing from ATRT regimens and focal radiotherapy. The outcome for many patients is a period of prolonged stability, but 4 deaths among 15 reported cases underscore the risk of malignant progression and the challenge of balancing under‐ and over‐treatment. Overall, the current literature and these two cases support maximal safe resection when feasible, close imaging follow‐up and treatment escalation at progression—often according to ATRT protocols—while avoiding overtreatment during the initial indolent phase.

## Author Contributions

AM and GAS drafted the manuscript and designed figures. PV supervised the study. MB, JMP and RS performed the molecular biology analyses. JP, AM, PL, LT and ABS collected the clinical data. Histological review was performed by AM, GAS, AV, ATE and PV. All authors critically revised the manuscript and approved the final version for submission.

## Funding

The authors have nothing to report.

## Ethics Statement

The authors have nothing to report.

## Consent

Written informed consent or nonobjection to participate in the study was provided by patients from GHU Paris Psychiatrie et Neurosciences and Hospices Civils de Lyon.

## Conflicts of Interest

The authors declare no conflicts of interest.

## Supporting information


**Figure S1:** Clinical and neuroimaging course of two LGDIT cases. Case #1 (top) presented with cognitive alterations. T1‐weighted with contrast enhancement (T1 w/ce) and FLAIR MRI at diagnosis show a bifrontal lesion with right predominance and contrast enhancement. Following biopsy, chemotherapy and radiotherapy, slow radiological progression occurred without clinical symptoms over 26 months of follow‐up. The patient, however, unfortunately passed away at 31 months. Case #2 (bottom) presented with tonic–clonic seizures. Initial MRI demonstrates a left parietal lesion with FLAIR and T1 w/ce. After surgical resection with intraoperative awake functional mapping, a small FLAIR‐hyperintense residual focus remained without contrast enhancement. The patient achieved good seizure control with anti‐epileptic drugs (AED), and the residual tumour remained stable at 24‐month follow‐up.

**Figure S2:** Immunohistochemical and fluorescence in situ hybridisation analysis of LGDIT cases. Case #1 (top) and Case #2 (bottom) show immunohistochemical stains with anti‐GFAP (glial fibrillary acidic protein), anti‐OLIG2 (oligodendrocyte transcription factor 2), anti‐CD34 (cluster of differentiation 34) and anti‐Ki‐67 (proliferation marker). Case #2 additionally demonstrates anti‐Vimentin positivity. Fluorescence in situ hybridisation (FISH) analysis of case #2, using ZytoLight SPEC *SMARCB1* dual‐colour probe (Clinisciences), shows *SMARCB1* loss (22q11, green) and *KREMEN1* (22q12, red) as a control.

**Table S1:** Compilation of LGDIT‐reported cases. CT: computed tomography; del: deletion; F: female; FU: follow‐up; ICE: ifosfamide, carboplatin, etoposide; IMRT: intensity modulated radiation therapy; M: male; mo: months; MTX: methotrexate; PBCST: peripheral blood stem cell transplantation; PD: progressive disease; rec: recurrence; TMZ: temozolomide; y: years.

## Data Availability

The data that support the findings of this study are available from the corresponding author upon reasonable request.
